# *Paeonia lactiflora* improves ovarian function and oocyte quality in aged female mice

**DOI:** 10.1590/1984-3143-AR2020-0013

**Published:** 2020-07-01

**Authors:** Min Jung Park, Si-Eun Han, Hyeon Jeong Kim, Jeong doo Heo, Hee-Jung Choi, Ki-Tae Ha, Sun Woo Yang, Kyu Sup Lee, Seung Chul Kim, Chang Woon Kim, Bo Sun Joo

**Affiliations:** 1 The Korea Institute for Public Sperm Bank, Busan, Repuplic of Korea; 2 Department of Obstetrics and Gynecology, Pusan National University School of Medicine, Busan, Repuplic of Korea; 3 Korea Institute of Toxicology, Jinju, Gyeongsangnam-do, Republic of Korea; 4 Healthy Aging Korean Medical Research Center, Pusan National University School of Korean Medicine, Yangsan, Republic of Korea; 5 Department of Obstetrics and Gynecology, Samsung Changwon Hospital, Sungkyunkwan University School of Medicine, Changwon, Korea, Republic of Korea

**Keywords:** ovarian aging, Paeonia lactiflora, ovarian function, oocyte quality

## Abstract

Although ovarian aging is a key cause of decreased ovarian function and oocyte quality, it remains a problem in infertility treatment. Therefore, this study is aimed to investigate whether *Paeonia lactiflora* (PL), a herb improves ovarian function and oocyte quality using aged female mice. C57BL/6 female mice aged 8 months were treated orally every day with PL of 26.5 mg/kg (n=7) and 53 mg/kg (n=7) of body weight for 4 weeks using an oral zoned needle. The control group (n=7) was treated with normal saline. Ovaries and serum were collected for the H&E stain and the evaluation of reactive oxygen species (ROS) levels, respectively. In the second experiment, female mice were orally administered with PL (26.5 mg/kg: n=12, 53 mg/kg: n=12, control: n=12) and then superovulated with PMSG and hCG, and mated with male mice. Zygotes were retrieved and cultured for 4 days. Ovaries were provided for examination of expressions of genes associated with angiogenesis (VEGF and visfatin), anti-aging (Sirt1 and Sirt2), and follicular development (c-Kit, BMP-15, and GDF-9). PL significantly increased numbers of surviving follicles (primordial, primary, secondary, and antral), numbers of zygotes retrieved, embryo development rate, and ovarian expression of VEGF, visfatin, c-Kit, BMP-15, and GDF-9 at both doses. However, ovarian expression of Sirt1 and Sirt2 was increased at 53.0 mg/kg of PL. ROS levels were not affected by PL. These results suggest that PL may possess beneficial effects regarding ovarian function and oocyte quality, possibly by activation of ovarian angiogenesis and follicular development.

## Introduction

Recently, age-related decline in fertility is becoming a significant issue in infertility treatment because more women are delaying marriage or childbearing. Advanced female age causes ovarian aging, finally resulting in decreased ovarian function and oocyte quality (Broekmans et al., 2007; Steiner et al., 2017; Duncan and Gerton, 2018). Age-related ovarian aging cannot be recovered and it remains a problem in infertility treatment. Nevertheless, several strategies have recently attempted to improve reproductive capacity with age (Ha et al., 2010; Choi et al., 2012; Celik et al., 2015; Özcan et al., 2016).

The deterioration of oocyte quality with age is mainly due to mitochondrial dysfunction via a decrease in cellular function of the oocytes (Thouas et al., 2005; May-Panloup et al., 2016). Oxidative stress is caused by mitochondrial dysfunction and finally induces aging of the cells, follicular atresia, and poor oocyte quality (Meldrum, 2013). It has been recently reported that Sirtuins, a representative anti-aging-related marker, are associated with gamete biology and reproductive physiology (Tatone et al., 2018). In this respect, the anti-oxidation and anti-aging have been proposed to improve age-related decline in oocyte quality (Bentov and Casper, 2013; Özcan et al., 2016).

As another reason for age-related decline in oocyte quality, decrease in ovarian angiogenesis has been suggested because ovarian angiogenesis plays an important role in a series of events of folliculogenesis (Geva and Jaffe, 2000; Fraser, 2006; Tatone et al., 2008). Some studies have demonstrated that the activation of ovarian angiogenesis can improve oocyte quality and ovarian function (Shimizu et al., 2003; Danforth et al., 2003; Choi et al., 2012). Angiogenesis is regulated primarily by various angiogenic factors such as vascular endothelial growth factor (VEGF), visfatin, and nitric oxide (NO) (Adya et al., 2008; Murohara et al., 1998). These factors play an important role in oocyte meiotic maturation and ovulation (Jablonka-Shariff and Olson, 1998). Although various factors regulating angiogenesis have been identified, there is no clinically effective treatment for improving oocyte quality in aged female.

The root of *Paeonia lactiflora* Pall. (belong to Paeoniaceae) is a herb that has traditionally being used in the treatment of gynecological disorders such as dysmenorrhea and infertility in oriental medicine of East Asian countries (Gao and Bernie, 1997; Jia et al., 2006). Our previous study showed that aqueous extract of *P. lactiflora* (PL) affects embryo implantation via increasing leukemia inhibitory factor-mediating endometrial receptivity (Choi et al., 2016). A combination of PL and *Glycyrrhiza glabra* extract was effective in normalizing menstrual cycles and hormonal balance in the patients with polycystic ovarian syndrome (PCOS) (Ushiroyama et al., 2001). Paeoniflorin extracted from PL extract improves ovulation in women with PCOS by reducing androgen production in theca cells (Ong et al., 2019). It also has various physiological effects including anti-inflammatory, anti-oxidation, and anti-aging (He and Dai, 2011; Chen et al., 2018).

However, there are no reports on the effects of PL on ovarian function and oocyte quality, especially for reproductive aged female. PL is being clinically used to treat gynecological problems including infertility in oriental medicine at a dose of 16 g per day for adult women weight 60 kg. This dosage is about 53 mg/kg, considering 20% of recovery rate of PL extraction. Therefore, this study determined 53 mg/kg and half as 26.5 mg/kg as the treatment concentration of PL, and investigated whether oral administration of PL improves ovarian function and oocyte quality in aged female mice.

## Methods

This study was approved by the Institutional Review Board of Pusan National University Hospital, Korea. All animal experiments were conducted under the guidance for the Care and Use of Laboratory Animals of the National Institutes of Health, approved by the Pusan National University Hospital Institutional Animal Care and Use Committee (Approval number: PNUH-2019-148).

### Animals

This study used inbred C57BL/6 mice in all experiments. The mice were purchased from the Koatech Inc. (Gyunggi-do, Korea) and bred under a 12 hours light/dark cycle with free access to water and food in an animal facility of SPF class with a temperature of 21±2 °C and the relative humidity of 55%±10%.

### Preparation of PL

The PL used in this study was an identical sample prepared with our previous study (Choi et al., 2016). Briefly, the roots of *P. lactiflora* were purchased from Omniherb Co. (Daegu, Korea) cultivated field in Gyeongsangbuk-do Province, Korea in 2013. The plant was identified by a botanical expert working at Omniherb (Daegu, Korea). A boucher specimen (DC-H21) was authenticated by HPLC analysis with several standard compounds including gallic acid, catechin, methyl gallate, paeoniflorin, and benzoic acid, and deposited in Korean Medical Research Center for Healthy Aging, Pusan National University (Busan, Korea). The roots of *P. lactiflora* (100 g) were extracted with 1 liter of distilled water for 2 hours at 100 ºC, and then centrifuged at 4000 rpm for 10 minutes.

### Treatment of PL

C57BL/6 female mice aged 8 months were randomly divided into three groups (n=7 per group): one control group and two PL treatment groups. PL was administered orally every day using syringes with an oral zoned needle at doses of 26.5 mg/kg and 53.0 mg/kg of body weight/150 μL for 4 weeks. The control group was treated with the same volume of normal saline as PL treatment.

### Measurement of body weight and ovarian weight

Body weights of animals were measured daily from the start of the experiment to just before sacrifice after 4 weeks of PL treatment. At the time of sacrifice, ovaries were collected, and weighed. The ovary weight index was defined by the following formula: ovary weight index=ovarian weight/body weight.

### Ovary and serum collection

At the time of sacrifice, after both ovaries were carefully removed and cleaned of fat tissue, the blood was collected from heart puncture using a syringe and needle and centrifuged at 1000 g for 15 minutes. And then the collected sera were stored at -80 °C until evaluation of ROS levels. The collected ovaries were weighed. Freshly dissected ovaries were divided as follows: One of the two ovaries from each mouse was immediately fixed in 4% paraformaldehyde for histopathology study, whereas the remaining tissues were immediately frozen at -80 °C until used for analysis.

### Histological Hematoxylin and Eosin (H&E) staining and ovary follicle counting

After 4 weeks of PL treatment, ovaries were fixed in 4% paraformaldehyde at 4 °C overnight, and dehydrated using ethanol series, cleared in xylene, embedded in paraffin, and sectioned for H&E staining. Sections were observed under a light microscope. Every section from each ovary was used for follicle counting and the results were corrected for double counting. Follicles were classified as an oocyte surrounded by a partial or complete layer of squamous granulosa cells into primordial follicle (an oocyte surrounded by one layer of flattened granulosa cells), primary follicle (an oocyte surrounded by one layer of cuboidal granulosa cells), secondary follicle (two or three layers of cuboidal granulosa cells with no antral space), and antral follicle (more than four layers of granulosa cells with one or more independent antral spaces, or with a cumulus granulosa cell layer)(Luo et al., 2008). Atretic follicles were considered the presence of apoptotic bodies in the granulosa cell layer, disorganized granulosa cells, a degenerating oocyte, or fragmentation of the oocyte nucleus (Liu et al., 2015).

### Superovulation, zygotes collection, and embryo culture

In the second part of the study, 36 female mice were administered orally every day with PL of 26.5 mg/kg and 53.0 mg/kg of body weight/150 μL for 4 weeks using syringes with an oral zoned needle. The day after the final administration of PL, the mice were superovulated by intraperitoneal injection with 0.1 mL of 5 IU pregnant mare’s serum gonadotropin (PMSG; Sigma-Aldrich, St Louis, MO, USA) followed by injection of 5 IU of human chorionic gonadotropin (hCG; Sigma-Aldrich) approximately 48 hours later. Then the mice were immediately paired with an 8-12-week-old individual male. The following morning the mice were inspected, and those with a confirmed vaginal plug were considered fertilized. Eighteen hours after hCG injection, female mice with a confirmed vaginal plug were killed by cervical dislocation, and cumulus-enclosed one-cell embryos (zygotes) were retrieved from the oviductal ampulae and denuded by incubation for 1 minute with 0.1% hyaluronidase (Sigma-Aldrich) in PBS (Giboc BRL, Grand Island, NY, USA). Zygotes were pooled and washed three times in G-IVF-plus medium (Vitrolife, V. Frolunda, Sweden) with 10% serum substitute supplement (SSS; Irvine, Inc. Santana, USA). Healthy zygotes only were cultured in 20-μL drops of Gl-plus medium (Vitrolife) with 10% SSS for the first 2 days, and then G2-plus medium (Vitrolife) with 10% SSS for the latter 2 days under paraffin-oil at 37 °C in a 5% CO2 incubator, and the media were changed daily.

### Expression of genes associated with angiogenesis, anti-aging, and follicular development in ovarian tissues

Just after the retrieval of the zygotes, both ovaries of each mouse were collected and mRNA expressions of genes associated with angiogenesis [vascular endothelial growth factor (VEGF) and visfatin], anti-aging [Sirtuins-1 (Sirt1), and Sirt2], and follicular development [Kit ligand receptor (c-Kit), bone morphogenetic protein-15 (BMP-15), and growth differentiation factor-9(GDF-9)] were measured by quantitative real-time PCR.

### Quantitative PCR

Total RNA was extracted using Trizol reagent (Invitrogen, Carlsbad, CA, USA) according to the manufacturer's protocol. Complementary DNA was synthesized from 1μg of total RNA using AMV Reverse Transcriptase (Promega, Madison, WI, USA) and a random hexamer (Takara Bio, Inc., Otsu, Japan) at 42 °C for 1 hour by inactivation of the enzyme at 95 °C for 5 min. Real-time PC R was performed using TOPreal™ qPCR 2X PreMIX SYBR (Enzynomics, Daejeon, Korea). Reaction mixtures were prepared using TOPreal™ qPCR 2X PreMIX, 0.5 pmol/μL of each primer, 100ng of cDNA, and sterile water (RNase free). The reaction conditions consisted of denaturation at 95 °C for 10 minutes, followed by 30 cycles of 95 °C for 10 seconds, 60 °C for 30 seconds. Each cDNA was subjected to polymerase chain reaction (PCR) amplification using gene-specific primers (Table 1). Quantitative PCR (qPCR) was carried out using LightCycle 480 SYBR Green I Master (Roche Diagnostics, Mannheim, Germany). The reaction conditions consisted of denaturation at 95 °C for 10 min, followed by 40 cycles of 95 °C for 10 s, 56 °C for 5 s, and 72 °C for 20 s. All experiments were performed in duplicate on each sample, eachbeing repeated at least three times. The relative expression levels of mRNA in each sample were calculated using the ddCt method. The level of each mRNA sample was normalized to the expression levels of the housekeeping gene GAPDH.

**Table 1:** Primers sequences used for real time PCR amplification.

**Gene**	**Sequence (5'→3')**	
	**Forward**	**Reverse**
VEGF	CT GTGCCTGCAGTGCGATAT	AGCTGCAGGTCCAGGATGTA
Visfatin	CTTGTTCAGTCCTGGTATCC	GCGAAGAGACTCCTCTGTAA
c-Kit	GCCTAGTCATTGTTGCA	TCCACCACCCTGTTGCTGTA
BMP15	TTGCTCCTCGTCTCTATACC	CTAGATGGCATGGTTGG
GDF9	GAGTGTGTTGACCATTGAACC	GCACCTCGTAGCTATCATGTC
Sirt1	TTGTGAAGCTGTTCGTGGAG	GGCGTGGAGGTTTTTCAGTA
Sirt2	AGCCAACCATCTGCCACTAC	CCAGCCCATCGTGTATTCTT
GAPDH	ACCACAGTCCATGCCATCAC	TCCACCACCCTGTTGCTGT

GAPDH, glyceraldehyde 3-phosphate dehydrogenase.

### Measurement of Reactive Oxygen Species (ROS) levels

Serum ROS levels were measured by the OxiSelect™ In vitro ROS/RNS Assay Kit (Cell Biolabs, Inc., Sandiego, CA) following the manufacturer's instructions. Briefly, 50 µL of sample and hydrogen peroxide standard was add to wells of a 96-well plate, and then 50 µL of Catalyst was additional added to each well. The wells were mixed and incubated for 5 minutes at room temperature. Then, 100 µL of DCFH solution was added to each well. The plate reaction wells were covered to protect them from light and incubated at room temperature for 15-45 minutes. The fluorescence of sample was read at480 nm excitation/530 nm emission with a fluorescence plate reader.

After ovaries were weighted quickly, the tissues were homogenized on ice with 0.8% ice-cold saline at a ratio of 1:9 (w/v). Then the homogenates were centrifuged for 10 min. Supernatants were collected for following detection. Total antioxidant capacity (TAC) was evaluated by quantitative colorimetric measurements in serum using the TAC Assay Kit (MyBioSource, San Diego, CA, USA) as per manufacturer’s instructions.

### Statistical analysis

An SPSS program (ver. 12.0) was used for statistical analysis. Statistical analysis for comparison of follicle counts, ovarian mRNA expression of each gene, serum ROS level, and the number of zygotes retrieved was performed by Student t-test. Embryo development rate to blastocyst were analyzed by one-way analysis of variance with post hoc multiple comparisons by Bonferroni-Dunn analysis. A P value of<.05 was considered statistically significant.

## Results

### Effect of PL on body and ovarian index

**Figure 1:**
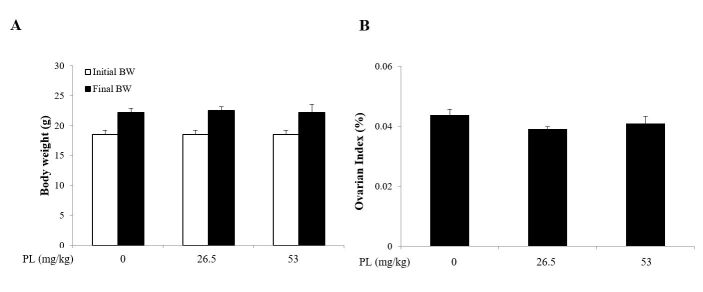
Effect of PL on body and ovary weights. After female mice were administrated 26.5, and 53 mg/kg of PL for 4 weeks, body and ovary were weighed, and the data was presented as the mean ± SD. (A) The body weight of mice in each groups (n = 7); (B) The ovarian index (n = 7). Ovarian index were calculated as ovarian weight/final body weight just before sacrifice. BW: body weight.

The body weights of all mice were recorded each day from the start of the experiment to just before sacrifice after 4 weeks of PL treatment. No changes in eating, drinking, fur color, and exploratory behavior has been observed during PL treatment. The body weight was increased normally and no difference was observed between control and PL-treated groups (p > 0.05; Figure 1A). The ovary weight and the organ index of ovary were also not significantly different between the control and treated groups (Figure 1B).

### PL improved ovarian function in natural ovarian aging mice

**Figure 2:**
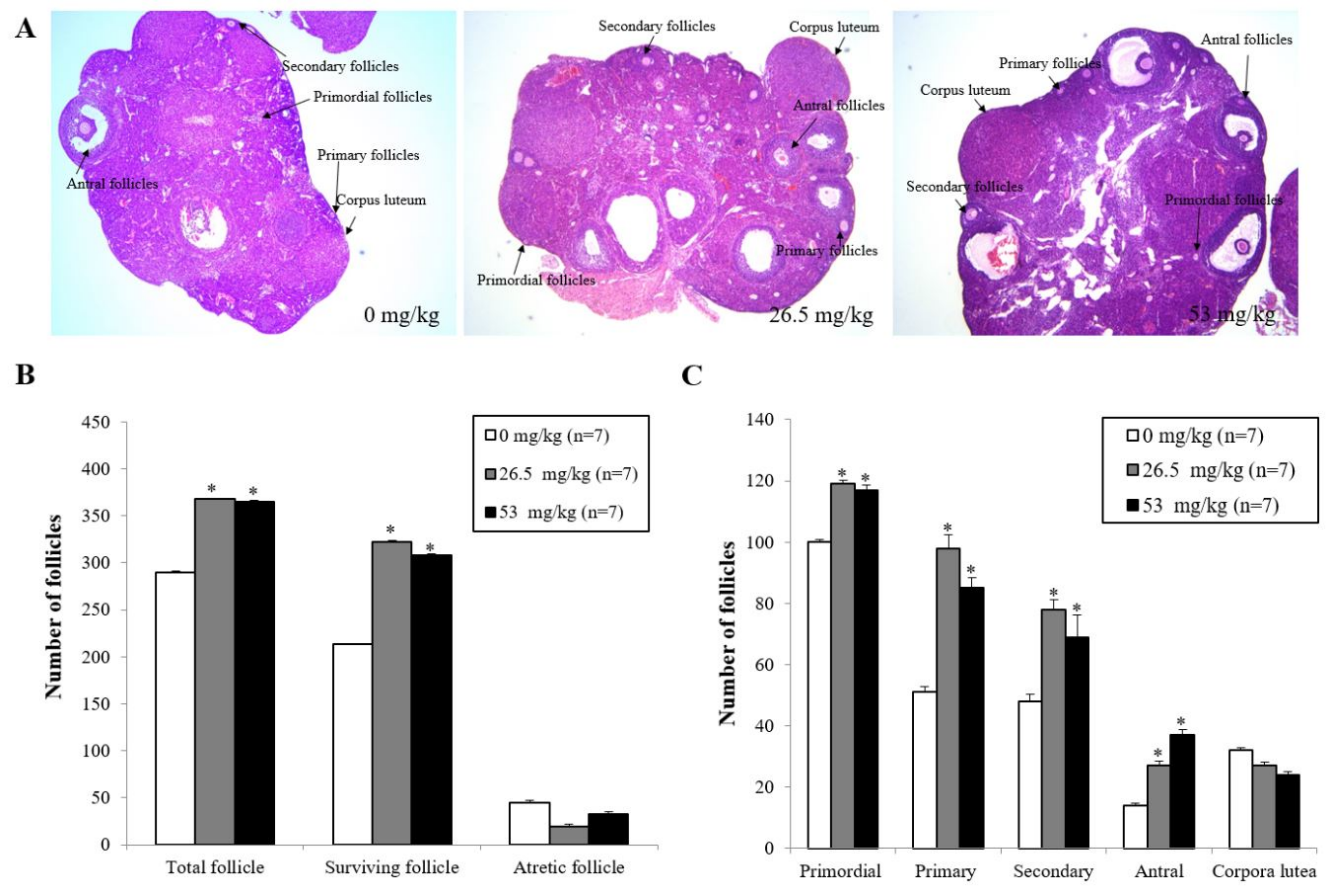
Effect of PL on follicular development. (A) Representative hematoxylin and eosin stained histological images of mouse ovary after water-extracted PL; (B) Comparison of the total number of follicles, surviving follicles, and atretic follicles; (C) Numbers of primordial, primary, secondary, and antral follicles. Data for the number of each follicle were the mean ± SD of all samples of each mouse. *P<0.05 (vs control group).) (n = 7 each group).

The histological characteristics of follicles at each stage after PL treatment were shown in Figure 2A and mainly composed of surviving follicles (primordial, primary, secondary, and antral follicles), corpora lutea, and atretic follicles. The total number of follicles was defined as the sum of the number of all stages of follicles in one ovary. The H&E stained ovarian tissues showed that PL increased the total number of follicles (368±1.4 at 26.5 mg/kg and 365±2.2 at 53.0 mg/kg) compared with the control group (290±0.8) with an increase rate of 26.9% at 26.5 mg/kg and 25.9% at 53.0 mg/kg. Of these, numbers of surviving follicles in the PL treatment group were 322±1.2 at 26.5 mg/kg dose and 308±2.9 at 53.0 mg/kg dose, which was significantly more than that of the control group (213±0.9). The increasing rates were 51.2% at 26.5 mg/kg and 44.6% at 53.0 mg/kg. In contrast, numbers of atretic follicles were 19±1.7 at 26.5 mg/kg of PL and 33±1.7 at 53.0 mg/kg dose, which was less than that of the control group (45±1.7), with d decrease rate of 48.9% and 26.7%, respectively (Figure 2B). The number of primordial follicles in the PL treatment group was 119 ± 1.1 at 26.5 mg/kg and 117 ±1.8 at 53.0 mg/kg, which was significantly more than that of the control group (100.0±0.8). The number of primary follicles in the PL treatment group was 98.0 ± 4.4 at 26.5 mg/kg and 85.0 ±3.2 at 53.0 mg/kg, which was significantly more than that of the control group (51.0±1.8). Numbers of secondary and antral follicles were significantlyincreasedjust at 26.5 mg/kg of PL (78.0 ± 3.2 and 27.0±1.6, respectively) compared to the control group (48.0±2.2 and 14.0±0.7). Especially, the number of antral follicles increased to almost double in the 26.5 mg/kg PL treatment. The number of corpora luteahad no significant difference was similar in control group and the PL treatment groups (Figure 2C).

PL significantly increased by about twice the number of zygotes retrieved to 21.5 and 22.3 at 26.5 mg/kg and 53.0 mg/kg, compared with 10.8 of control group (P<0.05). The embryo development rate to blastocyst was also significantly higher in PL treatment group (37.6% at 26.5 mg/kg and 30.8 at 53.0 mg/kg) compared to the control group (6.6%), but there was no significant difference between PL treatment groups (Table 2).

**Table 2:** Effect of PL on number of zygotes retrieved and embryo development.

**PL (mg/kg of BW)**	**No. of mice provided**	**No. of zygotes retrieved**	**Mean no. of zygotes retrieved /mouse**	**No. of zygotes fragmented (%)**	**No. of zygotes cultured**	**No. of 2-cell embryos (%)**	**No. of blastocysts (%)**
0	12	127	10.8±3.2	51 (40.2)	76	36 (47.4)	5 (6.6%)
26.5	12	263^a^	21.5±6.1a	42(16.0)^a^	221	144 (65.2)^a^	83(37.6)^a^
53.0	12	267^a^	22.3±3.5^a^	46 (17.2)^a^	221	135 (61.1)	68(30.8)^a^

^a^ *P*< 0.05 (vs controls). BW: body weight.

### PL increased ovarian expression of genes associated with angiogenesis, anti-aging, and follicular development

**Figure 3:**
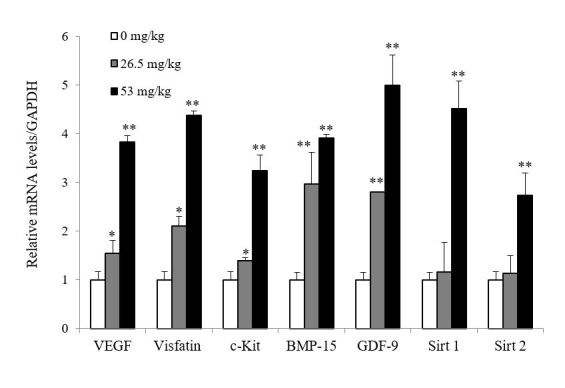
Expression of angiogenesis (VEGF and visfatin), anti-aging (Sirt1 and Sirt2), and folliculogenesis (c-Kit, BMP-15, and GDF-9)-related genes determined by quantitative real-time PCR. Whole ovaries were collected just after the retrieval of the zygotes. PCR was performed in duplicate on each sample. Relative gene expression levels were calculated versus GAPDH. Data are presented as mean±SD. *P<.05 (vs control); **P<.01 (vs control).

Expressions of VEGF and visfatin were significantly increased after PL treatment compared to the control group (P<0.05). In particular, expressions of these genes were further increased at dose of 53.0 mg/kg than at dose of 26.5 mg/kg (P <0.01). Also, PL treatment significantly increased the expression of c-Kit, BMP-15, and GDF-9, which have been well known to play important roles in follicular development and ovarian aging. Expressions of these genes were also higher at 53.0 mg/kg than at 26.5 mg/kg of PL (P <0.01). Expressions of BMP-15, and GDF-9 were relatively increased more than c-Kit expression. Expressions of Sirt1 and Sirt2 were significantly increased only at 53 mg/kg of PL (P <0.05) compared to the control group (Figure 3).

### Effect of PL on oxidative stress

**Figure 4:**
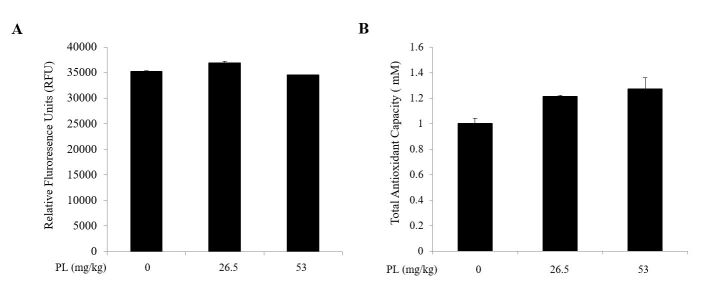
Effect of PL on reactive oxygen species (ROS) level. (A) Serum ROS production after 4 weeks of PL treatment. Data are given as Relative Fluorescence Units (RFU) ± SD of two duplicate processes in two separate experiments; (B) Change of total antioxidant capacity (TAC) in ovarian tissues after treatment of PL. Data are presented as mean±SD (n = 7 each group).

Serum ROS levels were similar among control and PL treatment groups regardless of dose of PL (Figure 4A). Total antioxidant capacity in the treatment of PL group was slightly increased compared with the control group, but revealed no significant difference (Figure 4B). It was suggested that PL had no effect on the generation or reduction of oxidative stress in aged ovary.

## Discussions

The present study showed that PL treatment increases not only the number of surviving follicle from primary follicles to antral follicles, but also the number of retrieved zygotes and embryo development competency. This study used female mice aged 8 months, which corresponds to the early 40s of humans (Age Converter, 2020). Since that age, the number of follicles and ovarian function begin to decrease rapidly. These results firstly demonstrated that PL can improve ovarian function and oocyte quality to withstand ovarian aging.

The exact mechanism for the beneficial effect of PL in aged mice remains to be elucidated. However, several possibilities can be considered. First is the activation of primordial follicles and ovarian angiogenesis by PL. The activation of dormant primordial follicles can continue to provide developing follicles and fertilizable oocytes for the entire reproductive lifespan (McGee and Hsueh, 2000). Many studies have suggested that ovarian function and oocyte quality can be improved through the activation of primordial follicles and ovarian angiogenesis (Ha et al., 2010; Celik et al., 2015; Li et al., 2010).

Ovarian angiogenesis plays a crucial role in a series of events of follicular development and follicular growth (Geva and Jaffe, 2000; Fraser, 2006). Indeed, increased vascular density is highest in mature follicles (Zelezni et al., 1981). Oocytes from poorly vascularized follicles developed in morphologically inferior embryos as compared to those from well-vascularized follicles, indicating that vascularization of the follicles can be a potential marker for the developmental potential of an embryo (Nargund et al., 1996). Dysregulation of VEGF may attenuate follicular or oocyte maturation by stimulating AMHR2 overexpression, which increases AMH binding (Fang et al., 2016). These results suggest that ovarian angiogenesis is very deeply involved in oocyte maturation process.

The activation of primordial follicles and follicular development are regulated by various intracellular signaling pathways and intraovarian factors such as VEGF, BMP series, GDF-9, and c-Kit (Paulini and Melo, 2011; Hsueh et al., 2015). Especially, GDF-9 and BMP-15 play a critical role in early follicular development and ovarian function (Paulini and Melo, 2011; Chang et al., 2016). As expected, the present study showed that PL increased the expression of VEGF, visfatin, GDF-9 and BMP-15, which were genes associated with angiogenesis and follicular development.

Second is the delay or restoration of aging itself by PL because some studies reported a potential activity of anti-aging of PL by scavenging active oxygen (Chen et al., 2018). Sirtuins has been well known to be associated with aging and longevity as a family of NAD^+^-dependent deacetylase (Vachharajani et al., 2016; Grabowska et al., 2017). Recently, it has reviewed that Sirtuins play an important role in gamete biology and reproductive physiology (Tatone et al., 2018). Among them, Sirt1 and Sirt2 are associated with oocyte maturation (De La Fuente, 2006; Luciano et al., 2014), and suppression of ovarian aging by preserving primordial follicles (Liu et al., 2013; Zhou et al., 2014; Chen et al., 2015). In this reason, we investigated changes in expression of Sirt1 and Sirt2 genes in the ovary after PL treatment. Their mRNA expression was significantly increased only by 53 mg/kg of PL, but at 26.5 mg/kg.

Third possible mechanism is the reduction of oxidative stress-induced ovarian aging by PL because some components of PL, such as anthocyanin and flavonoid, and phenolic compound, have antioxidant properties (Lee et al., 2017; Yang et al., 2018). In addition, quercetin, a bioflaconoid widely found in many leave and fruits, protects age-related decline in ovarian function by decreasing oxidative stress (Wang et al., 2018). However, the present study showed that ROS levels had no difference between PL treatment and control groups in serum or tissue samples, suggesting that PL in the present study had no antioxidant effect.

These results suggest that stimulation of ovarian angiogenesis and follicular development, and the inhibition of ovarian aging may increase oocyte quality, resulting in improvement of oocyte competence for embryo development. However, what was unexpected that improvement of oocyte quality and increase in the number of surviving follicles were slightly increased at 26.5 mg/kg of PL compared to 53.0 mg/kg without significance, whereas the expressions of genes associated with angiogenesis (VEGF and visfatin), follicular development (BMP-15, GDF-9), and anti-aging (Sirt1 andSirt2) were significantly increased at 53.0 mg/kg of PL compared to 26.5 mg/kg. This result means that the concentration on gene expression and the concentration on egg quality and follicle development through gene products are not necessarily parallel. This means that although gene expression is promoted at high concentrations, egg quality and follicular development can be sufficiently improved by gene products expressed at low concentrations.

The most difficult point in starting this study was to determine the treatment concentration of PL. To determine an optimal concentration, our study group previously tested the cytotoxic effect of PL on Ishikawa cells (Choi et al., 2016). PL did not show significant cytotoxicity up to a concentration of 100 μg/mL of high concentration. Second, PL is being clinically used to treat gynecological problems including infertility in oriental medicine at a dose of 16 g per day for adult women weight 60 kg for 4 weeks. This dosage is about 53 mg/kg, considering 20% of recovery rate of PL extraction. In several previous *in vivo* studies, PL was administered for 14-38 days (Lee et al., 2005; Li et al., 2012; Wu et al., 2014). Therefore, this study determined 53 mg/kg and half as 26.5 mg/kg as the treatment concentration of PF for 4 weeks. These concentrations are similar to the concentrations used in other study on the protection of liver injury by PL using mice which treated PF from 25 mg to 100 mg/kg body weight (Liu et al., 2006).

Another important value of this study is that overcoming ovarian aging can be an effective therapeutic strategy for menopausal symptoms. Hormonal replacement therapy is practically used to treat menopausal problems, but it has an increased risk of cancer or recurrence in cancer survivors. So, new researches are being needed on the development of effective method to delay or overcome natural ovarian aging and some studies have been attempted using various kinds of stem cells (Ding et al., 2018). Therefore, if PL in this study has the same effect in human studies, PL could be developed as an important remedy for solving menopausal problems.

However, one of the limitations of the present study is that this study did not reveal the critical components for the beneficial effect of PL. Likewise other plant materials, the components of *P. lactiflora* are variable by seasonal and regional changes (Kim et al., 2006; Oyungerel et al., 2017; Zhu et al., 2015). However, the Paeonia genus-specific glycosides, such as paeoniflorin, albiflorin, and 1,2,3,4,6-penta-O-galloyl-β-D-glucose, are most abundant compounds in most of crude drug samples collected from different regions or seasons (Kim et al., 2006; Parker et al., 2016; Zhu et al., 2015). Among them, paeoniflorin was reported that regulates the synthesis of steroid hormones in ovarian cells (Ong et al., 2019; Takeuchi et al., 1991). However, there are no previous studies reporting the effect of these compounds on the regulation of ovarian function and oocyte in aged mice. Therefore, further studies are needed to elucidate whether these compounds are involved in the improvement of ovarian function and oocyte quality by PL.

## Conclusions

The present study shows that water-extract PL improves ovarian function, oocyte quality and oocyte embryo development competency in aged female mice. In addition, PL increases ovarian expression of angiogenic factors (VEGF and visfatin), follicular development regulatory factors (c-Kit, BMP-15, and GDF-9), and anti-aging genes (Sirt1 and Sirt2) while ROS level was not affected by PL. These results suggest that the beneficial effects of PL may be attributed to the activation of ovarian angiogenesis and follicular development, and anti-aging event. These results may contribute to the development of PL as a new treatment strategy for ovarian aging or premature ovarian failure.
